# The dynamics of the aggressive order during a crisis

**DOI:** 10.1371/journal.pone.0232820

**Published:** 2020-05-22

**Authors:** Min-Young Lee, Woo-Sung Jung, Gabjin Oh

**Affiliations:** 1 Department of Physics, Pohang University of Science and Technology, Pohang, Republic of Korea; 2 Department of Industrial and Management Engineering, Pohang University of Science and Technology, Pohang, Republic of Korea; 3 Asia Pacific Center for Theoretical Physics, Pohang, Republic of Korea; 4 College of Business, Chosun University, Gwangju, Republic of Korea; 5 Lubar School of Business, University of Wisconsin-Milwaukee, Milwaukee, Wisconsin, United States of America; Universidad Veracruzana, MEXICO

## Abstract

We investigate the dynamics of aggressive order in the financial market to further understand volatility. To analyze aggressive order, market orders in the order book are scrutinized. The market orders have different degrees of aggressiveness; therefore, we categorize market orders into four types: types Zero, One, A, and B, of which type B is the most aggressive. To examine the dynamics and impacts of each type of order, we use both macro- and micro-level approaches. From the macroscopic perspective, the burstiness and memory of type B is highly correlated with volatility. When traders face a financial crisis, they place bursty aggressive orders, and the orders are more predictable than usual. From the microscopic perspective, we additionally focus on the influence of the orders, particularly the price impact and resilience. The aggressive order has a greater impact than others, even when the price change of the aggressive order is smaller. Moreover, the aggressive order delivers more information on price because the aggressive order has a higher price impact than the execution cost.

## Introduction

Volatility is an important indicator in the context of investment decisions, risk management, and monetary policy. Market participants and financial authorities use volatility as an indicator of risk. High volatility, especially during a crisis, can pose a great risk for investors and can confuse a market. A large number of researchers have studied volatility in a variety of ways. Many studies have been conducted to predict volatility using the ARCH and GARCH models. Santis [1] explained volatility from the dynamics of large price changes. There have also been attempts to deal with volatility from the levels of the trader and order book. Roll [2] analyzed the effect of the market microstructure on volatility. Alessio [3] adopted the agent-based model to show that the duration of limit order and the number of market order affect volatility. Figlewski [4] demonstrated that the bid-ask spread and tick size affect volatility. Næs et al. [5] showed that the slope of the order book is related to volume and volatility.

Many researchers have studied stylized facts to understand the statistics and dynamics of order books, for example, the memory of order flow [6–8], shape of order books, placement of an order [9], and characteristics of spread [10]. Because most traders want to minimize the impact of trading and the supervisory institution should control this impact, it is necessary to identify the price impact [11–21]. There has been a debate concerning the contradictory facts between the predictable order flow and unpredictable price due to price impact [16, 22]. Recently, Taranto et al. [23, 24] showed that the history-dependent impact model is superior to the transient impact model. Understanding the extreme and large event on the order book is also an active research field because an event greatly influences a market. There are several studies on the origin and dynamics of extreme and large events [11, 13, 25–31]. Farmer et al. [13] argued that a large trading volume does not always lead to a large price return and that the first gap equals the large return. Weber et al. [25] insisted that a large price change is induced by small liquidity and order flow. Corradi et al. [26] argued, in a small time scale, that the static depletion of the order book is related to the large price fluctuation. Meanwhile, Dufour et al. [32] shed light on the time and impact of transactions and verified that the decrease in the duration between transactions is related to the increase in the price impact and informed trader. There is a research trend of focusing on the interevent time between the specific events that have a greater impact on the market, rather than all transactions. Ren et al. [33, 34] analyzed the recurrence interval between trading volume and price return exceeding a certain threshold. They verified the short-term and long-term memory in the recurrence interval. Meng et al. [35] explained that the behavior of traders placing an order causes the interval distribution of the extreme event. Jiang et al. [30] used the hazard rate of the recurrence interval to predict the short-term extreme return.

Here, we explain the volatility using the dynamics of the aggressive order. The aggressiveness is the degree to which the participants take a risk of adverse price movement to trade immediately. The reason that we consider an aggressive order is that aggressiveness is more influential for market volatility than a price change itself, which will be verified. Although a large price change seemingly has a large impact on the order book, a large price change is not always proportional to the price impact and aggressiveness. Many people think a large price change will have a great impact on the order book, but in many cases, the impact might be small. In this case, the price changes seem to be too large. It is noise-like rather than a large change with a large impact. Rather, the aggressive order has a large impact on the order book, which we will verify.

This paper proposes a new perspective on the relationship between a large price change and aggressiveness. Our definition of aggressiveness is different from the previous criteria used by Biais et al. [36] and Large [37]. Because market orders have different degrees of aggressiveness, they are categorized into four aggressive types based on the adverse price movement mechanism: types A (jump), B (penetration), One (one tick change), and Zero (no price change). Type A jumps the first gap on the opposite side of the order book and has a small impact on price. Type B penetrates the opposite side and has a large impact. Aggressiveness differs even when the price change is the same for type A and B. Type One is a control that causes the price to change by one tick. Type Zero is also a control and does not cause any price change.

We apply both macro- and microlevel approaches to understand the dynamics of aggressive orders and the effects of an individual order. Aggressive order dynamics are related to market volatility from a macroscopic point of view, and the impact and information of an individual order are investigated from a microscopic point of view. The data we use consists of order books from 65 firms in the London Stock Exchange (LSE) from August 1, 2008 to March 31, 2009.

Macroscopic analysis shows that traders use a strategy of submitting aggressive orders, explaining volatility using aggressive order dynamics. The distribution of interevent time (IET) of aggressive orders follows a fat-tailed distribution, which means that aggressive orders also stem from trader strategy and are not random. After macroscopic analysis, the burstiness and memory in the IET of aggressive orders are calculated and compared with market volatility. The results show that the burstiness and memory coefficients of interevent time of aggressive orders sharply increased when the Lehman Brothers became bankrupt and slowly recovered from the crisis. In other words, traders submitted aggressive orders in a more bursty and predictable way when volatility was high during a crisis. To the best of our knowledge, this is the first study to analyze the relationship between the burstiness and memory of the interevent time of order and market volatility.

The microscopic approach investigates the impact of an individual aggressive order. It has been verified that price impact depends on aggressiveness, even if price changes are the same. To this end, we analyze the resilience and price impact from the price change before and after the transaction. Although the price change is small, the price impact of type B is greater than that of type A. We also consider imaginary profit by comparing the transaction cost and the long-term impact to confirm that the aggressive order has price information. Although the execution cost of type B is expensive, the imaginary profit of type B is higher than that of type A due to the large long-term impact, which means the former is more informative. Finally, we show that type B, the most aggressive order, has a significant impact on the order book and contains a large amount of price information. Type B is more likely to be submitted by institutional traders.

The contributions of this paper is as follows. First, market orders are investigated considering the adverse price movement. We confirm that the market orders with different levels of aggressiveness have a distinct characteristics. We describe market volatility as the burstiness and memory of an aggressive order. Furthermore, by analyzing price change and aggressive orders in detail, we reveal that aggressiveness is a major factor on price impact. Finally, we demonstrate that aggressive orders contain a large amount of price information.

## Notation and data

In a double auction market, a limit order is a type of order in which a liquidity provider chooses a price *p* and volume *V*. The liquidity provider submits the limit order, and he or she can cancel the order. A market order is a type of order in which a liquidity taker only chooses the volume *V* and wants to trade immediately at the available price on the market. If a trader submits a buy (sell) limit order at higher (lower) than a best ask (bid), this buy (sell) limit order is the same as a buy (sell)-initiated market order. This buy (sell) limit order is called an effective buy (sell)-initiated market order. A buy (sell) limit order at lower (higher) than a best ask (bid) is an effective buy (sell) limit order. In this paper, we use the definition of the effective market order and the effective limit order.

Best bid *b*_1_(*n*) is the highest bid price at the *n*th event time, and best ask *a*_1_(*n*) is the lowest ask price at the *n*th event time. The *n*th event time indicates the time point right before the *n*th transaction. Mid-price is the mean of the best bid and the best ask. $p\left(n\right)=\frac{1}{2}\left(a_{1}\left(n\right)\;+\;b_{1}\left(n\right)\right)$. In this study, a price difference is used instead of a return because we focus on a price change in the bid and ask side and use a condition of the change in a tick price. *d*_*a*_(*n*) = *a*(*n*) − *a*(*n* − 1), *d*_*b*_(*n*) = *b*(*n*) − *b*(*n* − 1). The difference between the best ask and the best bid is the spread. *s*(*n*) = *a*_1_(*n*) − *b*_1_(*n*). The second-best bid (ask) price is the second highest (lowest) price, and the first gap refers to the price difference between the best bid (ask) price and the second-best bid (ask) price. *g*_*b*,1_(*n*) = *b*_1_(*n*) − *b*_2_(*n*), *g*_*a*,1_(*n*) = *a*_2_(*n*) − *a*_1_(*n*). The *k*th gap refers to the price difference between *k*th best bid (ask) price and (*k* + 1)th best bid (ask) price. *g*_*b*,*k*_(*n*) = *b*_*k*_(*n*) − *b*_*k*+1_(*n*), *g*_*a*,*k*_(*n*) = *a*_*k*+1_(*n*) − *a*_*k*_(*n*). We introduce a tick consumption, which is how many ticks on the order book a market order consumes. If a buy (sell)-initiated market order takes all of the limit order in the best ask (bid) and does not take all of that in the second best ask (bid), *c*_*a*_ equals 1 (*c*_*b*_ equals 1). *c*_*a*_ = *x*(*c*_*b*_ = *x*) means that a buy (sell)-initiated market order takes all of the limit order up to the *x*th best ask (bid) and does not take all of that in the (*x* + 1)th best ask (bid). The reason for using tick consumption is that the price change traders can see in a market is directly related to tick consumption. A positive event is related to a buy-initiated market order, and a negative event is related to a sell-initiated market order.

We analyze the order book data of the London Stock Exchange (LSE). All of the information on the limit order, market order, and cancellation is recorded in the time resolution of one millisecond. The LSE comprises the on-book market and off-book market. This study only deals with the on-book market data. The on-book market is a fully automated electronic market, where traders submit their orders to the order book, and there is no official market maker such as a specialist in the NYSE. Because the LSE has no official market maker, the market participants should keep the market liquid, which is associated with resilience. We use the order book of the top 65 firms, and the main criterion of selection is the highest market capitalization. The firms included were those that did not change their ISIN (International Securities Identification Number), that were continuously traded in the period of our data, and that were traded in the British Pound. The period of our data is from August 1, 2008, to March 31, 2009. The data of September 8, 2008, are excluded because the LSE upgrades a trading platform for 7 hours; therefore, the number of trading days used is 168. The LSE opens at 8:00 and closes at 16:30. We only include the continuous trading time from 8:00 to 16:30, exclude the uncrossing period before and after the continuous trading and exclude the after-hours trading. The data of the first 100 transactions and the last 100 transactions are excluded in a day to remove the effect of the opening and closing market.

The list of abbreviations of 65 firms is arranged according to the market capitalization on August 1, 2008: HSBA, BP, VOD, RDSA, GSK, RIO, RDSB, BG, AAL, BATS, BLT, AZN, RBS, XTA, TSCO, BARC, DGE, STAN, IMB, RB, ULVR, LLOY, BA, NG, SAB, PRU, ENRC, AV, BT, SSE, CNA, EMG, CBRY, SKY, KAZ, MRW, CPG, RR, IPR, LAND, ABF, LGEN, TLW, SBRY, VED, ANTO, PSON, OMU, SN, SLA, UU, RSA, CPI, MKS, CW, SMIN, 0EV1, BLND, JMAT, INTU, NXG, SVT, KGF, HMSO, and AMFW.

## Methods

### Burstiness memory

Goh et al. [38] introduced burstiness ($B_{0}=\frac{r-1}{r+1},r=\frac{\sigma }{\mu }$; *μ* is the mean of the IET and *σ* is the standard deviation of the IET), which captures the intermittent feature, and the memory coefficient (*M*), which captures a temporal correlation between two consecutive IETs. Kim et al. [39] introduced an alternative burstiness measure that removes the size effect of the time series.

$$\begin{array}{c} B_{1}\left(r\right)=\frac{\sqrt{n+1}r-\sqrt{n-1}}{\left(\sqrt{n+1}-2\right)r+\sqrt{n-1}},\;r=\frac{\sigma }{\mu }. \end{array}$$ (1)

$$\begin{array}{c} M=\frac{1}{n-1}\sum_{i=1}^{n-1}\frac{\left(\tau_{i}-\mu_{1}\right)\left(\tau_{i+1}-\mu_{2}\right)}{\sigma_{1}\sigma_{2}}. \end{array}$$ (2)

where *μ* and *σ* are the mean and standard deviation of the IET, respectively. *τ* is the time series of the IET, and *n* is the length of the IET.

### Resilience & price impact

To quantify the resilience, the conditional average of the variables is calculated using time lags. The event time scale is considered, rather than a real-time scale, because the trading activity in real time is not uniform. The event time increases by one when the price or the volume of the best price change. This definition of the event time is different from that of the IET, where the event was a transaction. When focusing on a certain trading event, the event becomes the reference point. The lag equal to zero indicates the right before the reference point. The time lag of 100 events is used before and after the reference. We verify the robustness of the results by using different time lags of 10, 20, 50, and 200 event times. To compare the bid or ask price from the different levels and different stocks, we use the tick price rather than the real price and set the trading price to zero. If there is a transaction in the ask side, the best ask price is set to zero, which is the relative tick price.

$$\begin{array}{c} G_{A}\left(\tau |\Delta \right)=E\left[A\left(n+\tau \right)|a_{1}\left(n+1\right)-a_{1}\left(n\right)=\Delta \right] \end{array}$$ (3)

*G*_*A*_ is a response function of variable *A*, conditioned on the ask price change Δ. *E*[*A*(*t*)] refers to the average *A* over time *t*. *A*(*t*) is a value of A right before time *t*.

To quantify the resilience and impact that the aggressive order has, we adopt the concepts of a long-term impact and a reversion introduced by Ponzi et al. [40]. They found the linear relation between the immediate and permanent impact. A transaction makes an immediate impact, and the initial impact recovers. When a buy-initiated market order increases the best ask price, the bid (ask) price immediately increases, and the spread increases. After the immediate impact, the bid (ask) reverts to the state of recovery for several ticks.

$$\begin{array}{c} I_{A}\left(\tau \right)=A\left(n+\tau \right)-A\left(n\right)=\Delta A_{0}+R_{A}\left(\tau \right) \end{array}$$ (4)

*I*_*A*_(*τ*) is a long-term impact of variable A after time *τ*. The long-term impact is defined as the difference between *A*(*n*) and *A*(*n* + *τ*), where *A*(*n*) is the value of *A* right before *n*th event. Δ*A*_0_ = *A*(*n* + 1) − *A*(*n*) is an immediate impact and *R*_*A*_(*τ*) = *A*(*n* + *τ*) − *A*(*n* + 1) is a reversion. To calculate the average of a reversion and a long-term impact, it needs the data of infinite time by definition. We just use the proxy due to the empirical study and set *τ* as 100 event time scales and verify the robustness by using different time lags 10, 20, 50, 200 event times.

$$\begin{array}{c} E\left[R_{A}\left(\tau \right)|\Delta a_{0}\right]\equiv E\left[A\left(n+\tau \right)-A\left(n+1\right)|\Delta a_{0}\right] \end{array}$$ (5)

The average reversion (*E*[*R*_*A*_(*τ*)|Δ*a*_0_]) refers to an ensemble average and time average of the variable *A* conditioned on the difference of the ask price.

## Definition of aggressive order

A transaction cost includes the explicit and implicit cost. The explicit cost indicates the fees or taxes charged by a broker and the exchange. The implicit cost includes the market impact cost, and a timing and opportunity cost. Gomber et al. [41] divided the market impact cost into a liquidity premium related to spread and the adverse price movement. Aggressiveness is the degree to which a market participant risks market impact and reduces the risk of timing and opportunity costs. Griffiths et al. [42] stated that an aggressive order is associated with an adverse selection and has a low opportunity cost.

The following statement seems correct: A large price change is caused by an aggressive order and will have a great impact on the price. A change in price, however, is not always proportional to the aggressiveness or the price impact. Additionally, the impact of a large price change is not always large. In fact, the impact depends on aggressiveness, not on the price change itself. This concept will be verified in the section on resilience and price impact. To this end, we define aggressiveness based on how adverse a price change is considering the transaction cost. The adverse price movement is a price change that a participant does not want in order to minimize transaction costs. In this paper, we focus on the mechanism involved in making an adverse price movement because it is related to aggressiveness and significantly affects the price impact.

Biais et al. [36] divided aggressiveness based on the order position related to the prevailing bid-ask price. The categories include all orders such as the limit order, market order, and cancellation. In this paper, we only analyze transactions made by an effective market order based on the assumption that traders might choose to absorb the market impact cost rather than the cost of timing and opportunity during the crisis. Although a market order is more aggressive than a limit order, we categorize a market order into four types of aggressive orders based on the mechanism of an adverse price movement because the role of the aggressive order becomes more significant during a crisis.

**Fig 1:**
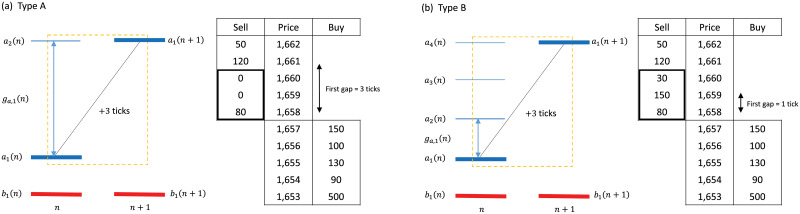
Explanation of order types. At the event time *n* and *n* + 1, the bid and ask, and the price change is the same for both cases. Type A is the event here price change equals to the first gap (*c*_*a*_ = 1 or *c*_*b*_ = 1) and (*d*_*a*_ > 1 or *d*_*b*_ > 1). Type B is the event where price change is larger than the first gap (*c*_*a*_ > 1 or *c*_*b*_ > 1) and (*d*_*a*_ > 1 or *d*_*b*_ > 1). The market order of type A makes the difference of the ask price *d*_*a*_(*n*) = 3 and the market order of type B makes the same price change.

We conjecture that aggressive orders have a larger impact than less aggressive orders of the same price change, and this conjecture will be verified in the section on resilience and price impact. The market orders are grouped into four categories, as shown in Fig 1. Even if the same price change occurs, the aggressiveness can differ. We show that types A and B make a price change larger than one tick. Type A consumes all of the opposite best and does not consume all of the second best (*c*_*a*_ = 1 or *c*_*b*_ = 1), and the tick change is larger than one (*d*_*a*_ > 1 or *d*_*b*_ > 1). This order jumps the first gap in the opposite side of order book and has a small price impact that we will verify later. Type B is the most aggressive order that consumes several ticks on the opposite side (*c*_*a*_ > 1 or *c*_*b*_ > 1). This order penetrates the opposite side by several ticks and has a large price impact. The reason for the criteria of tick consumption (*c*_*a*_ or *c*_*b*_) is that traders are sensitive to a price change and the variation in ticks. In comparison with type A, type B is the more aggressive order. Type One is the order that makes a price change of one tick (*c*_*a*_ = 1 or *c*_*b*_ = 1) and (*d*_*a*_ = 1 or *d*_*b*_ = 1). Type Zero is a control group that does not make any price change (*d*_*a*_ = 0 or *d*_*b*_ = 0). All market orders are classified as type Zero, One, A, or B. The most aggressive orders of Biais’ are mainly classified as type One and type A, which can be noise-like in the small time scale. Although type One and type A can consume the part of the volume of the second best, the execution cost that we will introduce later is almost zero. Many large price changes on a small time scale are due to type A, as in the previous study [13]; however, price changes are also due to type B in the crisis period.

**Fig 2:**
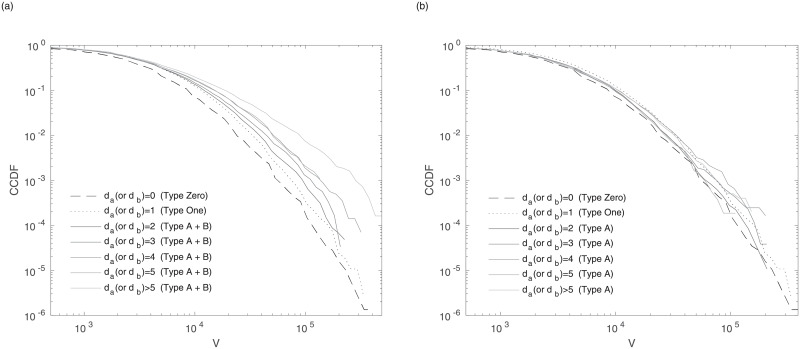
CCDF of trading volume(*V*) of HSBC. The distributions are conditional to the price difference (*d*_*a*_(or *d*_*b*_) ≥ 0) for (a) type A + type B, and (b) type A. As a control group, type Zero(dashed line) and type One(dotted line) are used.

Fig 2 is the distribution of trading volume conditional to a price change of HSBC. Each line indicates a price change *d*_*a*_(or *d*_*b*_). In the cumulative distribution of the nonzero tick price change, *d*_*a*_(or *d*_*b*_) = 1, 2, 3, 4, 5 correspond to the 69.09, 88.30, 93.86, 96.50, and 97.70 percentiles, respectively. For 65 firms, *d*_*a*_(or *d*_*b*_) = 1, 2, 3, 4, 5 correspond to similar percentiles on average. As shown in Fig 2(a), the volume distributions depend on the level of the price change. The distributions are more fat-tailed as the price change increases. The distributions of type A, however, as shown in Fig 2(b), are not dependent on the level of the price change and are even similar to those of type Zero and type One, which is a finding that is in line with the previous research [13]. Therefore, the dependency of volume on a price change in Fig 2(a) is due to type B. The tail of volume distributions follows a single power-law curve after the normalization, in which the volumes are divided by the standard deviation of the volume. The tail of the normalized volumes for the positive price change of all firms follows a power law with exponent 3.59 ± 0.73. The result of a power-law volume distribution is consistent with that of previous studies [43, 44]. The difference between this study and the previous study [13] where they showed the independency of volume on the price change is the period. The states of the market have changed during the crisis. In the crisis period, there are more aggressive tradings in the market. Alessio [3] also showed that the volatility is affected by the number of market order. The most aggressive order, type B, seems to be influenced by the state of the market because the number of type B significantly increases during the crisis as in S2 Fig. Meanwhile, as shown in Fig 2, the volume of type B is larger than that of types A, One and Zero. Because there have been many studies [45–48] that have argued that institutional traders submit more volume than individual traders, we think it is likely that institutional investors submit type B more than individual traders.

## Burst and memory of order with market volatility

Based on the definition of aggressive orders, we study the dynamics of the order and the effects of the individual order from a macro and micro perspective. In this section, we first identify the characteristics in the distribution of interevent time. In addition, volatility will be explained by the interevent time of aggressive orders on a macroscale. To this end, we must define the event and the interevent time.

The event is defined as a transaction and occurs when a trader submits a market order. Other traders can submit a limit order and cancellation between two consecutive trading events. The time interval between two consecutive trading events is called the interevent time (IET). Only the IET of intraday is considered, and the interday interval is excluded. The IET is closely related to the strategy where a trader determines the timing to submit order. S1 Fig is an illustration of the IET of HSBA as an example. The distribution of the IET is well fitted in the Weibull distribution, as shown in S1 Table. Unlike an exponential distribution from the Poissonian statistics, the Weibull distribution is fattailed, which means that the IET is not randomly distributed. The other firms are also well fitted in the Weibull distribution and the lognormal distribution. The lognormal distribution has a fat tail that follows the non-Poissonian statistics. The fat-tailed distribution of the IET is in accord with previous studies [49, 50]. Barabasi [51] also showed that the IET of human behavior is power-law-distributed and insisted that human behaviors are governed by a non-Poissonian process. The Weibull distribution of type B demonstrates that traders submit the most aggressive orders using a specific, rather than random, strategy.

The burstiness calculates the deviation of the IET from the Poisson process, meaning that there is a period of rapid submission and a calm period. *B*_0_(*r*) = 1 means that the IET is spike-like, and *B*_0_(*r*) = −1 means that the IET is uniform. If a process follows the Poissonian statistics, the IET has an exponential distribution and has *B*_0_(*r*) = 0. Because the lengths of the time series of the IET varies, as shown in S2 Fig, and can affect the result, we use the alternative burstiness (*B*_1_) measure to remove the size effect of time series, as shown below [39]. Burstiness of approximately 0.3 and Weibull distribution mean that trading activity is intermittent due to traders’ strategy. The memory coefficient, on the other hand, is related to the clustering of orders. For each trader, a high memory coefficient means that he or she wants to conceal the intention, and so, he or she splits the orders [52, 53]. Unfortunately, it is impossible to identify the IDs of traders in our LSE dataset. A high memory coefficient, therefore, cannot confirm such order splitting and can result from the herding behavior of traders.

**Fig 3:**
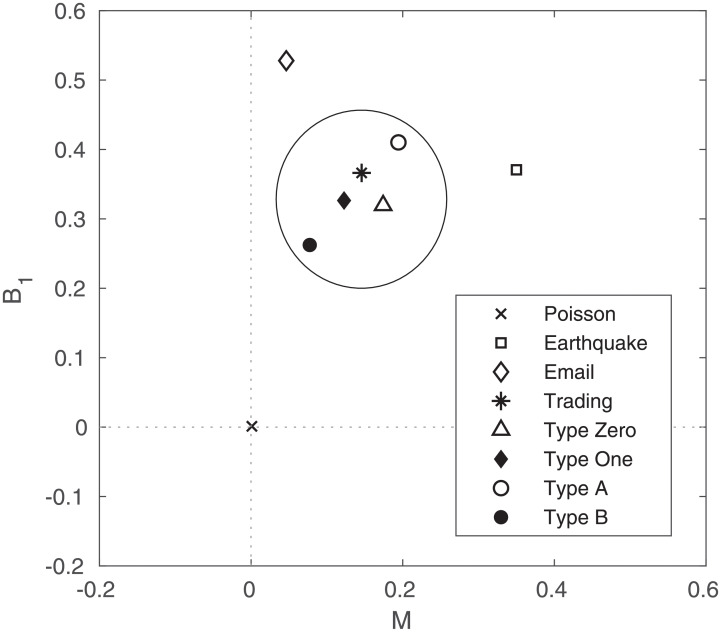
Averaged alternative burstiness and memory coefficient. The trading events are divided into type Zero(triangle), type One(filled diamond), type A(open circle) and type B(filled circle). The burstiness and memory coefficient are averaged for all firms. Earthquake(square), Email(diamond), and Trading(asterisk) of all types. Poisson process(cross) is a control group.

Fig 3 represents the burstiness (*B*_1_) and memory (*M*) of each type compared with the Earthquake [54], Email [55], and Poisson process. Goh et al. [38] explained that human behaviors have a low memory coefficient and moderate burstiness. The transaction (large circle in Fig 3) is also on the region explained by the behavior of humans and is not random. In this paper, we want to verify the aspects of the change in the burstiness and memory in the IET using a moving window with a length of 10 days.

**Fig 4:**
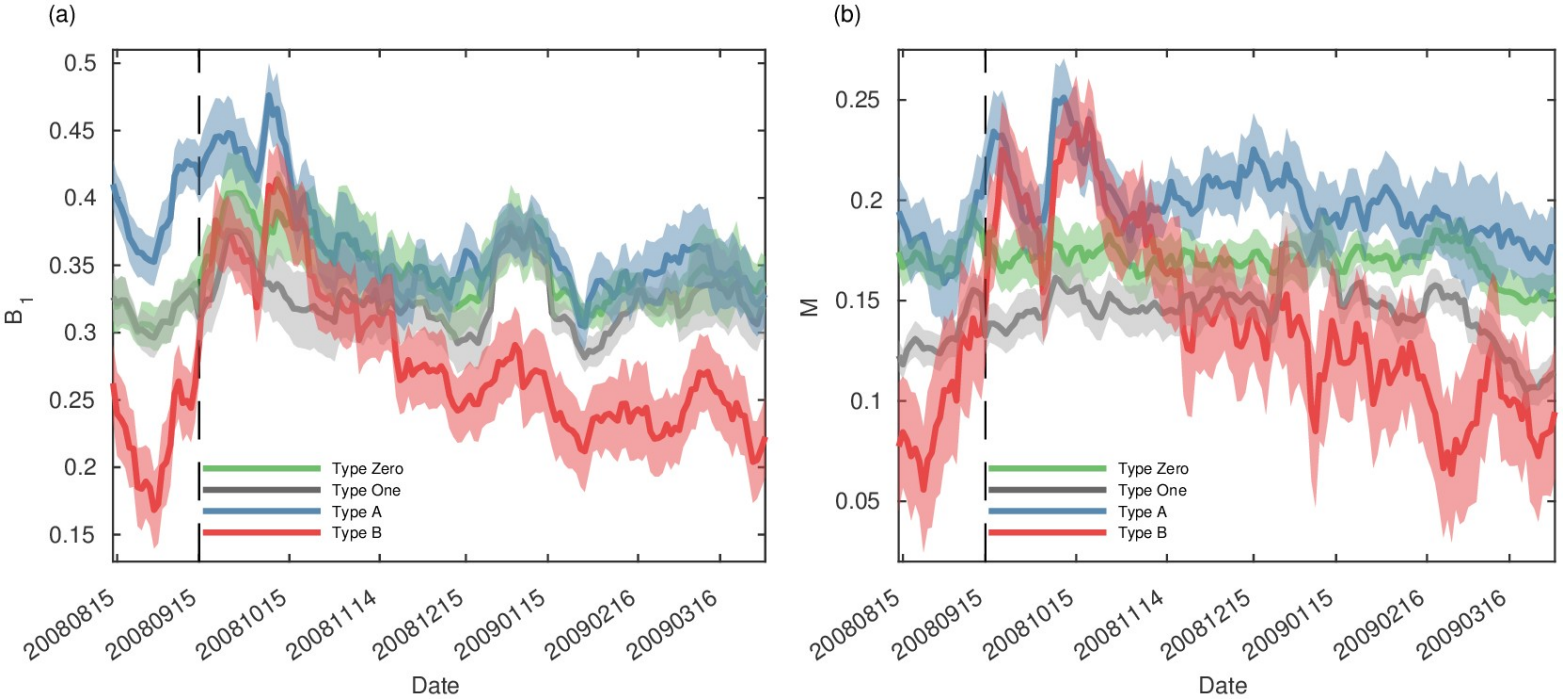
Time series of (a) the alternative burstiness *B*_1_ and (b) memory coefficient *M*. Type Zero(green), One(gray), A(blue) and B(red). The size of window is 10 days and the moving size is 1 day. The time series is averaged for all firms. The shaded areas indicate the 95% confidence level. The black dotted line refers to September 15th, 2008 when the Lehman Brothers became bankrupt. The two-sample Kolmogorov-Smirnov tests also show the statistically significant differences, especially at type B, between before, during, and after the crisis at the 1% significant level.

On September 15, 2008, Lehman Brothers became bankrupt, and the financial crisis affected the world, including London. The coefficients of the Pearson correlation between the S&P 500 and FTSE and VIX and FTSE are 0.59 and -0.58, respectively. In addition to the Pearson correlation, the Granger causality test supports that the S&P 500 Granger-causes the FTSE and that the VIX Granger-causes the FTSE. The burstiness and memory of the IET of the transaction, especially in type B, increase during the crisis period, as shown in Fig 4. The black dotted line is the point of the Lehman Brothers bankruptcy. After the minimum point on August 29, 2008, the burstiness and memory coefficient increased and showed a rapid change on September 15, 2008. To analyze the significant difference, the total period is divided into four periods using a window of 30 days. The period of the first 30 days is the ‘before crisis’ period, the 30 days immediately after the bankruptcy of Lehman Brothers is the ‘during crisis’ period, and the period of the final 30 days is ‘after crisis’ period. The two-sample Kolmogorov-Smirnov tests show statistically significant differences, especially for type B, before, during, and after the crisis at the 1% significance level. The distributions of the burst and memory coefficients during the crisis are significantly different from those in the before and after crisis periods. It is right-skewed, and there is more correlation between two consecutive events during the crisis. In other words, traders abruptly submit aggressive orders in some period and take the calm period, and it is easier to predict when other aggressive orders are placed in the future. For that reason, we argue that burstiness and memory can be used as the early warning signal as in the previous study [30]. To quantify this relationship in Fig 4, the Pearson correlation coefficients are calculated, as shown in Table 1. When all market orders are considered, burstiness and memory have a low correlation with volatility indexes such as the standard deviation of return and VIX and TED spread. Type One has a negative correlation with all volatility indexes. This implies that a price change by one tick becomes bursty when the volatility is low rather than high. On the other hand, the bursting behavior and memory coefficient of type B are significantly correlated with the volatility indexes. The analysis on the IET reveals how the bundle of events affect market volatility on the macroscale. We also identify the Pearson correlation coefficient between burstiness (*B*_1_), memory (*M*) and volatility (*M*) for individual firms. The statistics for individual firms show that type B has a statistically significant higher value than type A, type One, and type Zero, as shown in S3 Fig.

**Table 1:** Pearson correlation (left) between the burstiness and the volatility measures and (right) between the memory coefficient and the volatility measures. *V*_1_ is the mean volatility. *V*_2_ is the TED spread. *V*_3_ is the VIX. All types is the sum of type Zero, One, A, and B. The correlation coefficient is calculated using window size and moving size equal to 1 day. P-value of Pearson correlation. *:0.05, **: 0.01, ***: 0.001.

IET measure	Burstiness			Memory		
Volatility	V 1	V 2	V 3	V 1	V 2	V 3
All types	-0.0304	-0.0792	-0.1932 (*)	-0.2222 (**)	0.0440	-0.0429
Type Zero	0.2482 (**)	0.2585 (***)	0.1030	-0.1928 (*)	0.1253	0.1003
Type One	-0.3380 (***)	-0.2132 (**)	-0.3621 (***)	0.0352	0.2952 (***)	0.3001 (***)
Type A	0.0252	0.2204 (**)	-0.3033 (***)	0.2293 (**)	0.3818 (***)	0.2728 (***)
Type B	0.5263 (***)	0.7293 (***)	0.4094 (***)	0.5058 (***)	0.6900 (***)	0.4459 (***)

## Resilience of bid and ask around transaction

The microscopic approach investigates the effect of the individual order with aggressiveness. We will analyze the effect of individual event on the microscale and will argue how each type of event affects the bid-ask price using the concept of resilience and price impact. The conjecture introduced before, that the impact of type B is larger than that of type A, will also be verified. The price impact is closely related to the resilience of bid and ask prices around transactions.

Harris et al. [56] introduced the four dimensions that are associated with liquidity: width, depth, immediacy, and resiliency. Resilience is related to liquidity because a liquid market enables traders to exchange a stock without a large impact. Many authors have studied the dynamics of resilience [17, 40, 57–60]. They observed the recovery of the order book after the shock. Our paper also addresses the resilience of bid and ask around a transaction with aggressiveness.

**Fig 5:**
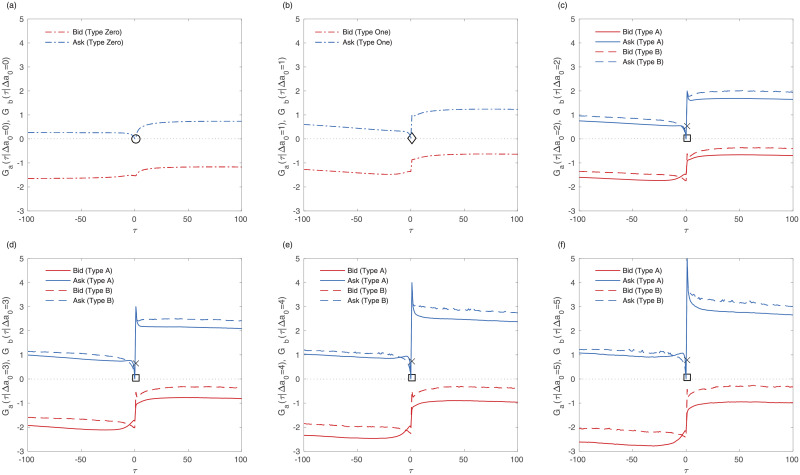
Resilience of the averaged bid and ask price around the positive event for all firms. The negative lag(*τ* < 0) means the pre-event time and the positive lag(*τ* > 0) means the post-event time. The zero lag(*τ* = 0) refers to the state right before the reference event of price change. The red line is the bid price and the blue line is the ask price. (a) Response function of the bid(red) and ask(blue) price to the event of price change(Δ*a*_0_ = Δ = 0). (b-f) Response function of the bid and ask price to the event of price change(1 ≤ Δ*a*_0_ = Δ ≤ 5). The circle, diamond, square, and cross mean the averaged execution tick cost of the type Zero, One, A and B. The zero execution tick cost means, for example, the buy initiated market order is traded at the opposite best ask price. The circle, diamond, and square are near the zero point meaning most of orders are traded at the opposite best price. The figures are arranged increasing the difference of ask price (*d*_*a*_ = Δ*a*_0_ = Δ) by one tick.

Even if the price changes are the same between types A and B, we suppose that the relaxation dynamics can be different. Fig 5 shows the resilience of the bid and ask price around a positive price change. The results of the negative event where a price goes down are the same as shown S4 Fig. Although the ask price changes are the same between types A and B, the resilience of type B is unlike that of type A. The change in the best ask of type B is larger than that of type A after the event. The best bid change around the event is also larger in the case of type B. Type B has more impact on the order book.

Ranaldo [61] explained that an eager trader submits a market order. He argues, however, that the eager trader does not refer to the informativeness of trader. To understand the information of the order, some authors have used numerical simulation [62]. In the empirical data, however, it is difficult to measure the information of the order. Griffiths et al. [42] explained the informativeness of the order using the excess return. The information of the order is closely related to the cost. When analyzed for cost [42], type B has a larger execution cost but a smaller opportunity cost than type A. We will analyze the information using the scheme of the cost and the excess price change. The execution costs of each type are marked as the circle, diamond, square and cross. The execution cost of type Zero is exactly zero. The fact that the execution cost equals zero means that the trader transacts at the opposite best price. The higher the execution cost is, the more traders should pay. The execution cost of type A is smaller than that of type B and is similar to that of type Zero. We will consider the informativeness of each type using the execution cost in the next section.

## Price impact and information

**Fig 6:**
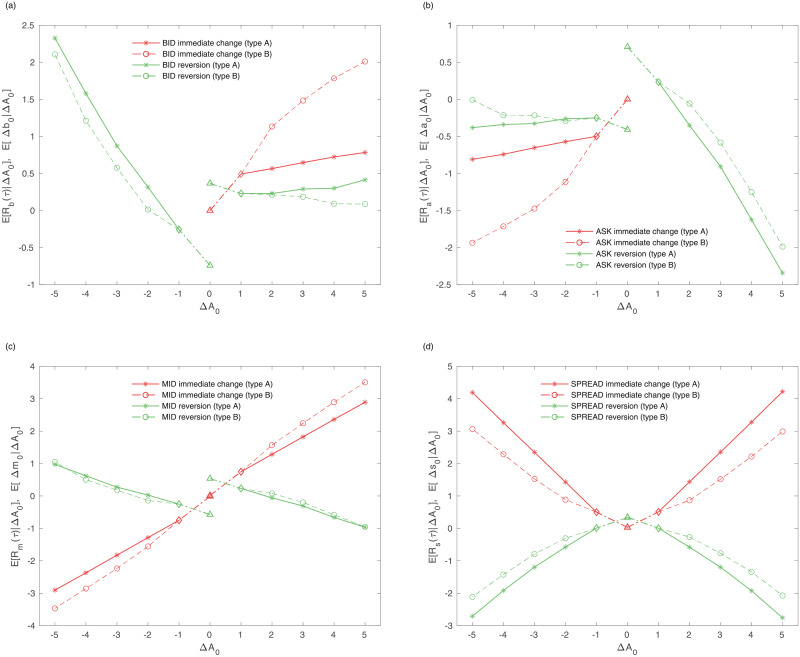
Immediate Impact(red line) and Reversion(green line) of (a) ask price, (b) bid price, (c) mid-price and (d) spread conditional to the immediate ask price change(Δ*a*_0_ = Δ). The impact and reversion are calculated by the resilience of all firms. For Δ*A*_0_ < 0 Δ*A*_0_ = −Δ*b*_0_, and for Δ*A*_0_ > 0, Δ*A*_0_ = Δ*a*_0_. The solid line is the type A and the dotted line is type B. The immediate impact and the reversion is calculated by the ensemble average in equation (6). The x-axis refers to the immediate change of the best price and the y-axis refers to the reversion and the immediate impact. The triangle, diamond, asterisk, and circle of symbols refer to type Zero and One, A, and B respectively.

**Fig 7:**
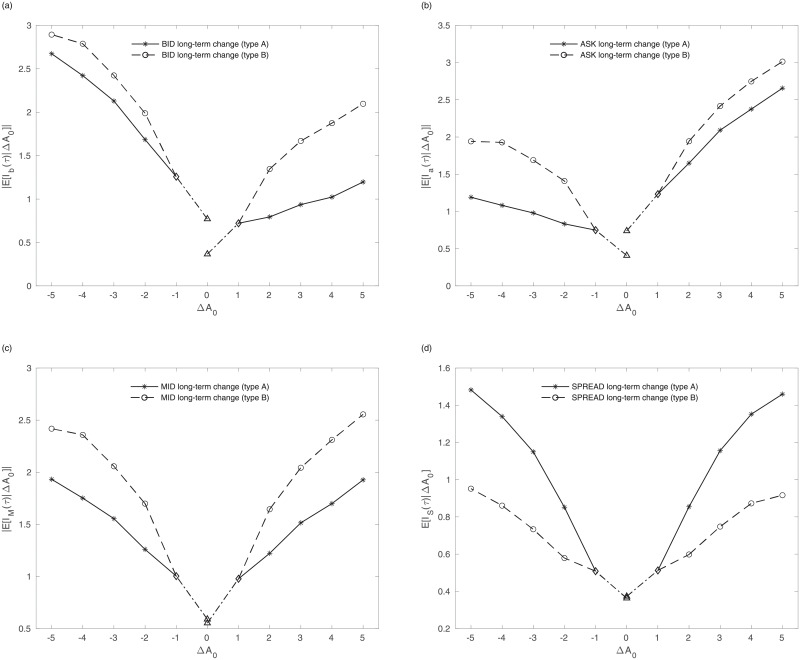
long-term Impact of (a) bid and (b) ask price, (c) mid-price and (d) spread conditional to the immediate price change(Δ*A*_0_ = Δ) in (6). The impact is calculated by the resilience of all firms. For Δ*A*_0_ < 0 Δ*A*_0_ = −Δ*b*_0_, and for Δ*A*_0_ > 0, Δ*A*_0_ = Δ*a*_0_. The solid line is type A and the dotted line is type B. The x-axis refers to the immediate change of the best price and the y-axis refers to the long-term impact. The triangle, diamond, asterisk, and circle of symbols refer to type Zero and One, A, and B respectively.

Following the approach in the previous section, the microscopic approach addresses the impact, cost, and information for an individual aggressive order. To quantify the resilience and impact that the aggressive order has, we adopt the concepts of long-term impact and reversion introduced by Ponzi et al. [40]. A transaction has an immediate impact, and the initial impact recovers. For example, when a buy-initiated market order increases the best ask price, the bid (ask) price immediately increases and the spread also increases. After the immediate impact, the bid (ask) price reverts to a state of recovery for several ticks. Even though the price recovers, it is not the same as it was before the impact—the difference is shown in the long-term impact. We show the immediate impact, reversion, and the long-term impact in Figs 6 and 7, respectively.

In Fig 6(a), we observe that type B induces the buy limit order in the spread. The negative slopes of the ask reversion, in Fig 6(b), indicate that the sudden jump in the ask recovers after the immediate change. Fig 6(c) and 6(d) are the immediate impact and the reversion of the mid-price and spread. The immediate change in the mid-price is larger in the case of type B, whereas the reversion of that is similar in the two types. The difference in mid-price comes from the immediate change rather than the reversion. The change in the spread is larger in the case of type A due to its noise-like characteristics. The long-term impacts of the bid and ask price increase with the absolute immediate changes shown in Fig 7. Type B induces a stronger impact than type A because the long-term impact of type B in the mid-price is larger than that of type A. Although a buy-initiated market order directly affects the ask side, the long-term impact is stronger on the bid side. Mainly due to the strong impact on the bid side, the mid-price impact of type B increases. The long-term impact of the spread increases with the change in the immediate ask, especially in type A, which means it is more stable.

Fig 5 and Table 2 represent the execution cost of type A and B. A trader who employs the type B order faces the disadvantage of execution cost, but he or she has the advantage of timing and opportunity cost. The execution cost of type A is almost zero even if the price change is large. The cost of type B, however, is larger than 0.5. To verify the informativeness of each type, we use the concept developed by Griffiths et al. [42]. We assume that traders should take the opposite position to make a profit. If a trader buys (sells) a stock, he or she makes a profit by selling (buying) a stock again. The difference between the execution cost and the long-term impact is used as the excess change. Therefore, the imaginary profit is calculated based on the long-term impact of the opposite position. We find that the imaginary profit of type B is larger than that of type A, type One, and type Zero. Because the long-term impact of the opposite side is larger than the execution cost, especially in type B, it is possible to say that type B has more information about the price than types A, One, and Zero.

**Table 2:** Execution cost and long-term impact of negative(upper table) and positive event(lower table). Imaginary profit is the difference between the long-term impact and execution cost. The imaginary profit of type B is larger than type A, One, and Zero. The imaginary profit increases as |Δ*A*_0_| increases. For Δ*A*_0_ < 0 Δ*A*_0_ = −Δ*b*_0_, and for Δ*A*_0_ > 0, Δ*A*_0_ = Δ*a*_0_.

Negative Δ*A*_0_	0	1	-2		-3		-4		-5	
Type	Zero	One	A	B	A	B	A	B	A	B
Execution cost	0.0000	0.0307	0.0338	0.5384	0.0439	0.6491	0.0556	0.7197	0.0694	0.7625
Long-term impact	0.4077	0.7494	0.8336	1.4093	0.9797	1.6901	1.0820	1.9280	1.1911	1.9419
Imaginary profit	0.4077	0.7187	0.7998	0.8709	0.9358	1.0410	1.0264	1.2083	1.1217	1.1794
Positive Δ*A*_0_	0	1	2		3		4		5	
Type	Zero	One	A	B	A	B	A	B	A	B
Execution cost	0.0000	0.0305	0.0335	0.5364	0.0442	0.6474	0.0558	0.7315	0.0679	0.7851
Long-term impact	0.3657	0.7213	0.7944	1.3455	0.9362	1.6692	1.0230	1.8745	1.1968	2.0966
Imaginary profit	0.3657	0.6908	0.7609	0.8091	0.8920	1.0218	0.9672	1.1430	1.1289	1.3115

**Fig 8:**
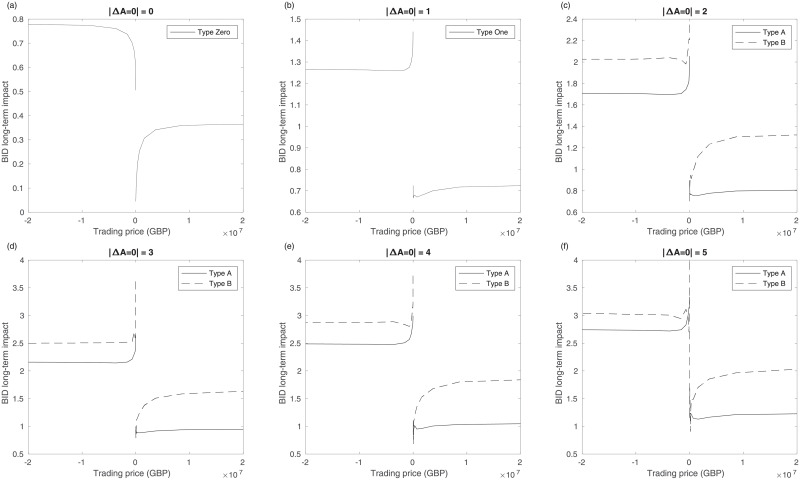
Mid long-term impact about trading price. Trading price is the trading volume multiplied by price and the unit is GBP. Solid line indicates type Zero, One, A and dashed line indicates type B.

Volume is another important factor in studying price impact. We focus on aggressiveness for several reasons. First, the greater the volume, the larger the price impact. For example, type Zero has a large impact when the volume is large. However, the same correlation does not hold for types One, A, and B. When the price decreases (increases), the opposite phenomenon appears in the bid (ask), as shown in Fig 8 and S5 and S6 Figs, where the long-term impact tends to decrease with volume when the volume is small. Fig 8 and S5 and S6 Figs represent the longterm impact of mid-price, bid, and ask, respectively. This result also has important implications in studying volume and price impact.

The total period is divided into 3 subsets: the period before (August 1, 2008–September 12, 2008) and during (September 15, 2008–October 27, 2008) and after (February 18, 2009–March 31, 2009) the crisis. As shown in S7 Fig, the spread during the crisis period is larger than before and after the crisis period, which means that the crisis period is more unstable. We verify that the impact is larger and that the order book structure is more unstable for all types in the period of crisis.

## Conclusion

In conclusion, we discuss the dynamics and price impact of an aggressive order. To this end, market order is categorized into four types: A (jump), B (penetration), One (one tick change), and Zero (no price change) based on the adverse price movement mechanism. The results show that the IET of the aggressive order describes the volatility well. Aggressiveness is the most important factor in analyzing market impact, and price change is next in importance.

The macroscopic result shows that the burstiness and memory of type B are highly correlated with volatility. In other words, traders submit more abrupt orders and then enter a calm period, making the temporal pattern of the orders more predictable due to such strategies used during a crisis. The relationship between the characteristics of the IET and volatility helps traders understand and even predict the market condition on the macroscale because traders only need the information in the order book that is open to everyone, especially type B information. A microscopic perspective also shows an individual order’s effect. To quantify the effect, we introduce the immediate impact, reversion, and long-term impact. Type B has a larger long-term impact, even if the price changes are smaller than type A’s. Type B contains more information about the price than others based on the difference between the execution cost and future price. Furthermore, the impact on spread shows that the market becomes more unstable during the crisis for all types. Because resilience and price impact results explain how the market reacts to each type’s market order, we can cope with market conditions, especially in a volatile market. This new perspective on a large price change and order aggressiveness broadens our understanding of volatility. This paper will also help researchers understand such information as it relates to order dynamics.

## Supporting information

S1 Fig Illustration about the IET of HSBA for (a) type Zero, (b) type One, (c) type A, (d) type B. The upper figures represent the 100 events in the real-time scale and the lower figures refer the complementary cumulative distribution function of IET. The solid lines represent each type and the dotted lines are the exponential distributions that have the same means. The statistics for the fitting is on S1 Table.
(PDF)

S2 Fig Timeseries of the number of type Zero, One, A, and B. The number of type Zero(black solid), type One(blue dotted), type A(green dashed), and type B(red dash dotted) increase after the bankruptcy of Lehman Brothers. The size of window is 10 days and the moving size is 1 day.
(PDF)

S3 Fig (a) Boxplot of Pearson correlation coefficients between alternative burstiness(B1) and volatility(v). Edges of the boxes refer the 25th and 75th percentiles. Plus symbols refer the averaged values. Type Zero—Type A has 0.05(*) significant, however, the marker is omitted. (b) Boxplot of Pearson correlation coefficients between memory coefficient(M) and volatility(v). Pvalue of KS-test. * :0.05, **: 0.01, ***: 0.001.
(PDF)

S4 Fig Resilience of the averaged bid and ask price around the negative event for all firms. The negative lag(τ < 0) means the pre-event time and the positive lag(τ > 0) means the post-event time. The zero lag(τ = 0) refers to the state right before the reference event of price change. The red line is the bid price and the blue line is the ask price. (a) Response function of the bid(red) and ask(blue) price to the event of price change(Δa0 = Δ = 0). (b-f) Response function of the bid and ask price to the event of price change(−5 ≤ Δa0 = Δ ≤ −1). The circle, diamond, square and cross mean the averaged execution tick cost of the type Zero, One, A and B. The zero execution tick cost means, for example, the sell initiated market order is traded at the opposite best bid price. The circle and square are near the zero point meaning most of orders are traded at the opposite best price. The figures are arranged increasing the difference of bid price (da = Δa0 = Δ) by one tick.
(PDF)

S5 Fig Bid long-term impact about trading price. Trading price is the trading volume multiplied by price and the unit is GBP. Solid line indicates type Zero, One, A and dashed line indicates type B.
(PDF)

S6 Fig Ask long-term impact about trading price. Trading price is the trading volume multiplied by price and the unit is GBP. Solid line indicates type Zero, One, A and dashed line indicates type B.
(PDF)

S7 Fig long-term Impact of spread conditional to the immediate ask price change(Δa0 = Δ) in equation (6) for the subdivided periods. The impact is calculated by the resilience of all firms. For ΔA0 < 0 ΔA0 = −Δb0, and for ΔA0 > 0, ΔA0 = Δa0. The solid line is type A and the dotted line is type B. 30 days before the crisis(BC, blue) and during the crisis 30(DC, red), and after the crisis(AC, green). The triangle, diamond, asterisk, and circle of symbols refer to type Zero and One, A, and B respectively.
(PDF)

S1 Table Statistics for fitting of the IET of HSBA. The distribution is fitted based on the criteria such as the negative log-likelihood, Akaike information criterion, Bayesian information criterion. Weibull distribution and Lognormal distribution are best fit for each type.
(PDF)
