# Supplementary material for: The dynamics of the aggressive order during a crisis
Source: PLoS One. 2020 May 22;15(5):e0232820. doi: 10.1371/journal.pone.0232820 (PMC7244114; doi:10.1371/journal.pone.0232820)
Supplement: S1 Fig — The upper figures represent the 100 events in the real-time scale and the lower figures refer the complementary cumulative distribution function of IET. The solid lines represent each type and the dotted lines are the exponential distributions that have the same means. The statistics for the fitting is on S1 Table. (PDF) [file pone.0232820.s001.pdf]

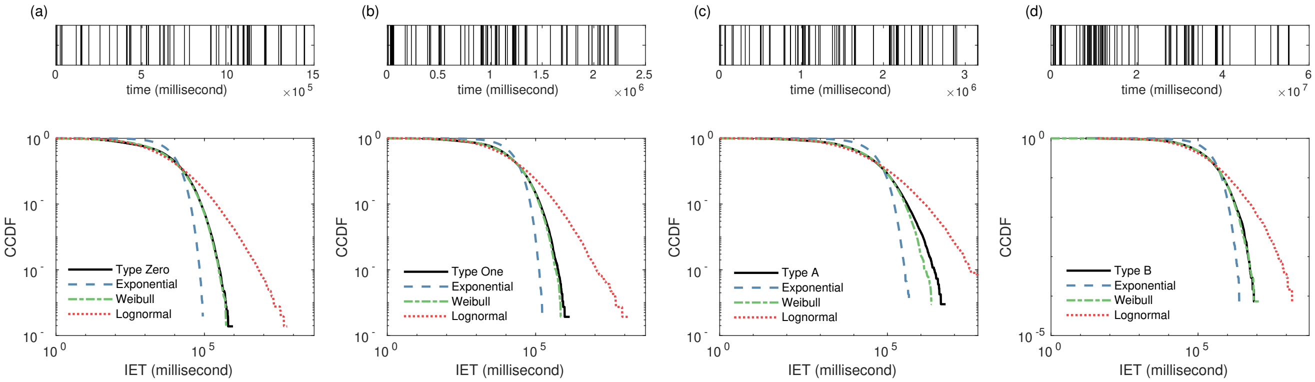

Figure S1. Illustration about the IET of HSBA for (a) type Zero, (b) type One, (c) type A, (d) type B. The upper figures represent the 100 events in the real-time scale and the lower figures refer the complementary cumulative distribution function of IET. The solid lines represent each type and the dotted lines are the exponential distributions that have the same means. The statistics for the fitting is on Supplementary Table.S1.
