# Supplementary material for: The dynamics of the aggressive order during a crisis
Source: PLoS One. 2020 May 22;15(5):e0232820. doi: 10.1371/journal.pone.0232820 (PMC7244114; doi:10.1371/journal.pone.0232820)
Supplement: S2 Fig — The number of type Zero(black solid), type One(blue dotted), type A(green dashed), and type B(red dash dotted) increase after the bankruptcy of Lehman Brothers. The size of window is 10 days and the moving size is 1 day. (PDF) [file pone.0232820.s002.pdf]

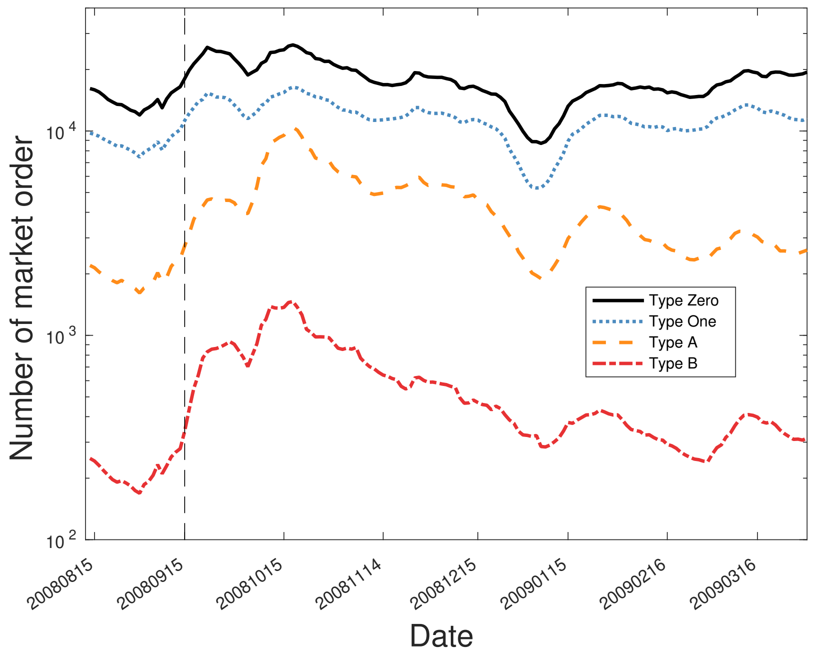

Figure S2. Timeseries of the number of type Zero, One, A, and B. The number of type Zero(black solid), type One(blue dotted), type A(green dashed), and type B(red dash dotted) increase after the bankruptcy of Lehman Brothers. The size of window is 10 days and the moving size is 1 day.
