# Supplementary material for: The dynamics of the aggressive order during a crisis
Source: PLoS One. 2020 May 22;15(5):e0232820. doi: 10.1371/journal.pone.0232820 (PMC7244114; doi:10.1371/journal.pone.0232820)
Supplement: S3 Fig — (a) Boxplot of Pearson correlation coefficients between alternative burstiness(B1) and volatility(v). Edges of the boxes refer the 25th and 75th percentiles. Plus symbols refer the averaged values. Type Zero—Type A has 0.05(*) significant, however, the marker is omitted. (b) Boxplot of Pearson correlation coefficients between memory coefficient(M) and volatility(v). Pvalue of KS-test. * :0.05, **: 0.01, ***: 0.001. (PDF) [file pone.0232820.s003.pdf]

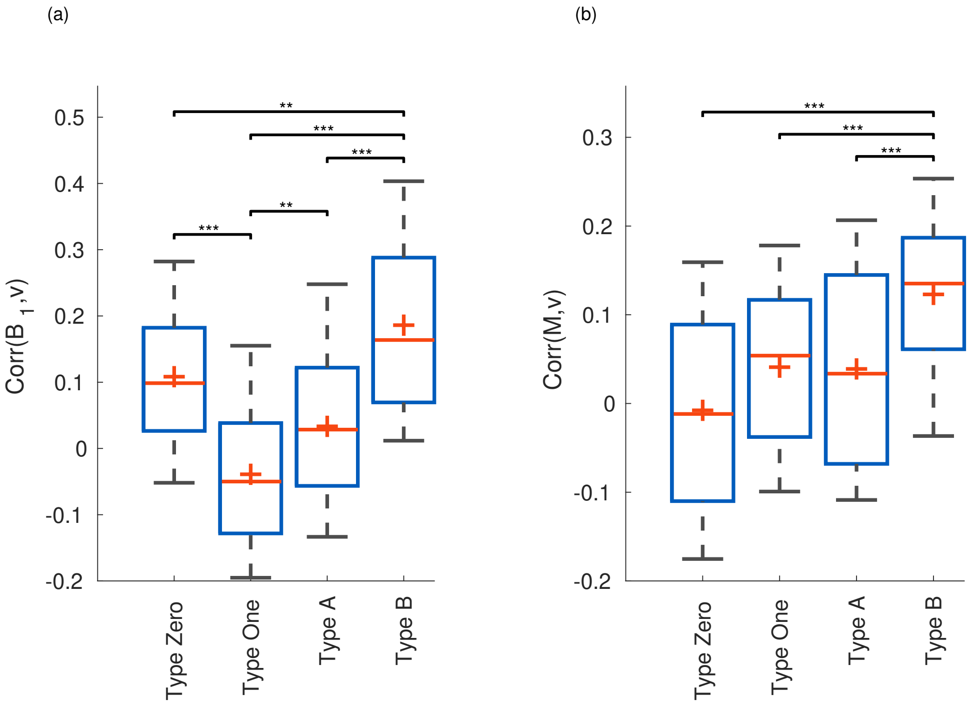


**Figure S3.** (a) Boxplot of Pearson correlation coefficients between alternative burstiness($B_1$) and volatility($v$). Edges of the boxes refer the 25th and 75th percentiles. Plus symbols refer the averaged values. Type Zero - Type A has 0.05(*) significant, however, the marker is omitted. (b) Boxplot of Pearson correlation coefficients between memory coefficient($M$) and volatility($v$). P-value of KS-test. * :0.05, **: 0.01, ***: 0.001
