# Supplementary material for: The dynamics of the aggressive order during a crisis
Source: PLoS One. 2020 May 22;15(5):e0232820. doi: 10.1371/journal.pone.0232820 (PMC7244114; doi:10.1371/journal.pone.0232820)
Supplement: S4 Fig — The negative lag(τ < 0) means the pre-event time and the positive lag(τ > 0) means the post-event time. The zero lag(τ = 0) refers to the state right before the reference event of price change. The red line is the bid price and the blue line is the ask price. (a) Response function of the bid(red) and ask(blue) price to the event of price change(Δa0 = Δ = 0). (b-f) Response function of the bid and ask price to the event of price change(−5 ≤ Δa0 = Δ ≤ −1). The circle, diamond, square and cross mean the averaged execution tick cost of the type Zero, One, A and B. The zero execution tick cost means, for example, the sell initiated market order is traded at the opposite best bid price. The circle and square are near the zero point meaning most of orders are traded at the opposite best price. The figures are arranged increasing the difference of bid price (da = Δa0 = Δ) by one tick. (PDF) [file pone.0232820.s004.pdf]

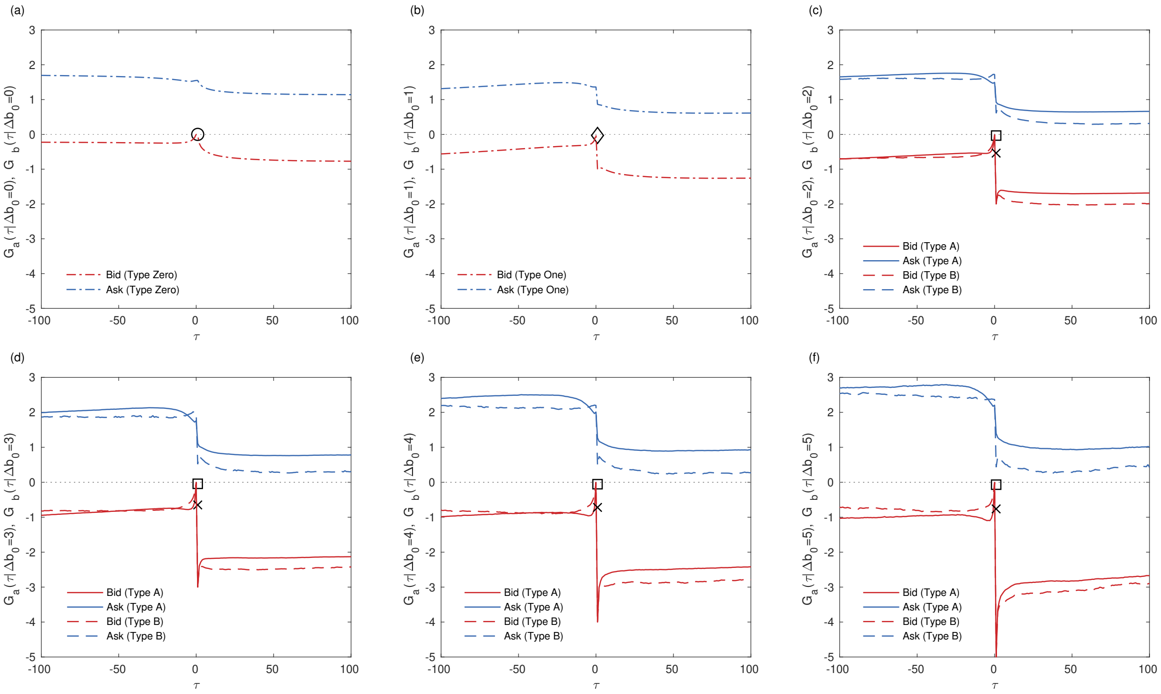

Figure S4. Resilience of the averaged bid and ask price around the negative event for all firms. The negative lag($\tau < 0$) means the pre-event time and the positive lag($\tau > 0$) means the post-event time. The zero lag($\tau = 0$) refers to the state right before the reference event of price change. The red line is the bid price and the blue line is the ask price. (a) Response function of the bid(red) and ask(blue) price to the event of price change($\Delta a_0 = \Delta = 0$). (b-f) Response function of the bid and ask price to the event of price change($-5 \leq \Delta a_0 = \Delta \leq -1$). The circle, diamond, square and cross mean the averaged execution tick cost of the type Zero, One, A and B. The zero execution tick cost means, for example, the sell initiated market order is traded at the opposite best bid price. The circle and square are near the zero point meaning most of orders are traded at the opposite best price. The figures are arranged increasing the difference of bid price ($d_a = \Delta a_0 = \Delta$) by one tick.
