# Supplementary material for: The dynamics of the aggressive order during a crisis
Source: PLoS One. 2020 May 22;15(5):e0232820. doi: 10.1371/journal.pone.0232820 (PMC7244114; doi:10.1371/journal.pone.0232820)
Supplement: S5 Fig — Trading price is the trading volume multiplied by price and the unit is GBP. Solid line indicates type Zero, One, A and dashed line indicates type B. (PDF) [file pone.0232820.s005.pdf]

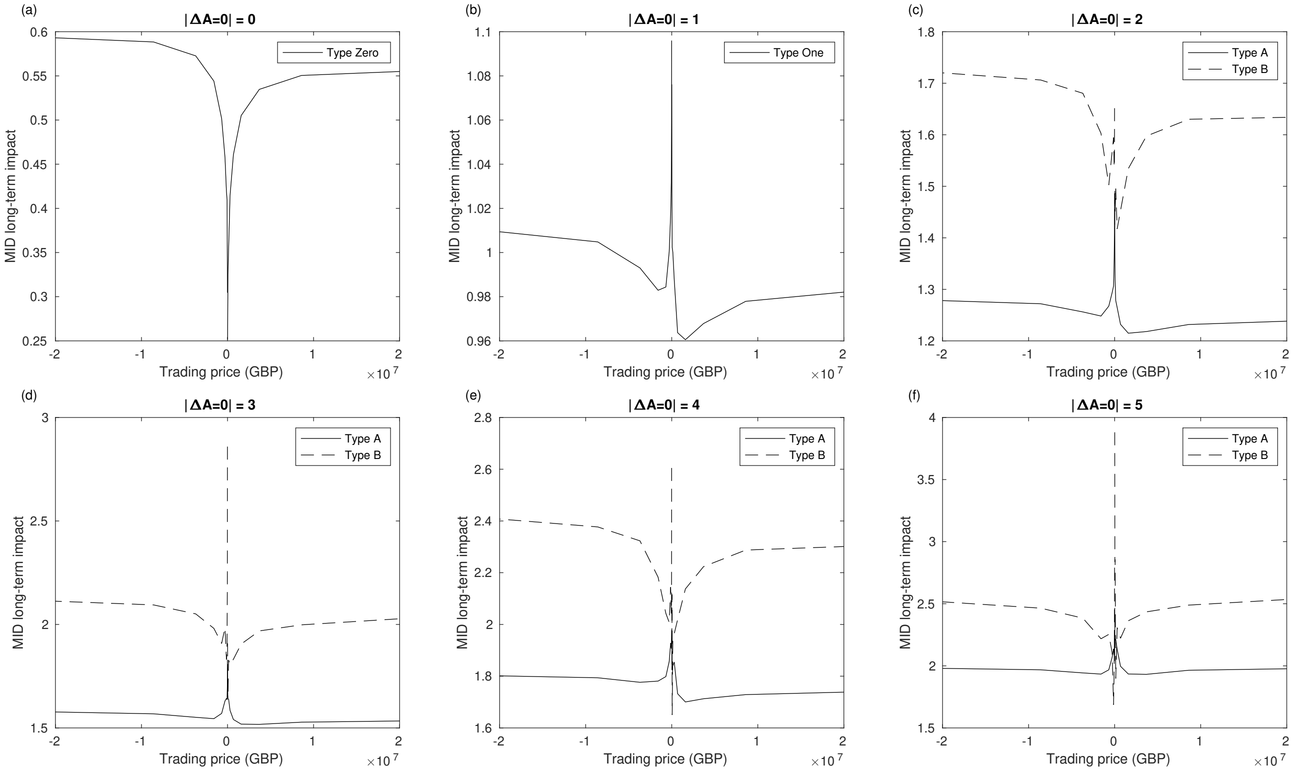

Figure S5. Bid long-term impact about trading price. Trading price is the trading volume multiplied by price and the unit is GBP. Solid line indicates type Zero, One, A and dashed line indicates type B.
