# Supplementary material for: The dynamics of the aggressive order during a crisis
Source: PLoS One. 2020 May 22;15(5):e0232820. doi: 10.1371/journal.pone.0232820 (PMC7244114; doi:10.1371/journal.pone.0232820)
Supplement: S7 Fig — The impact is calculated by the resilience of all firms. For ΔA0 < 0 ΔA0 = −Δb0, and for ΔA0 > 0, ΔA0 = Δa0. The solid line is type A and the dotted line is type B. 30 days before the crisis(BC, blue) and during the crisis 30(DC, red), and after the crisis(AC, green). The triangle, diamond, asterisk, and circle of symbols refer to type Zero and One, A, and B respectively. (PDF) [file pone.0232820.s007.pdf]

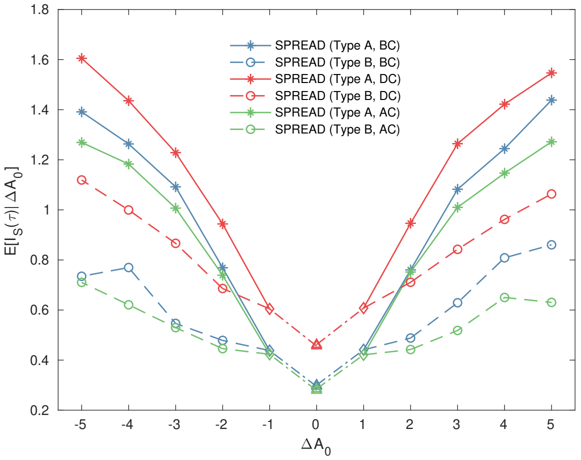

Figure S7. long-term Impact of spread conditional to the immediate ask price change($\Delta a_0 = \Delta$) in equation (6) for the subdivided periods. The impact is calculated by the resilience of all firms. For $\Delta A_0 < 0$ $\Delta A_0 = -\Delta b_0$, and for $\Delta A_0 > 0$, $\Delta A_0 = \Delta a_0$. The solid line is type A and the dotted line is type B. 30 days before the crisis(BC, blue) and during the crisis 30(DC, red), and after the crisis(AC, green). The triangle, diamond, asterisk, and circle of symbols refer to type Zero and One, A, and B respectively.
