# Supplementary material for: The dynamics of the aggressive order during a crisis
Source: PLoS One. 2020 May 22;15(5):e0232820. doi: 10.1371/journal.pone.0232820 (PMC7244114; doi:10.1371/journal.pone.0232820)
Supplement: S1 Table — The distribution is fitted based on the criteria such as the negative log-likelihood, Akaike information criterion, Bayesian information criterion. Weibull distribution and Lognormal distribution are best fit for each type. (PDF) [file pone.0232820.s008.pdf]

	Distribution	NlogL	BIC	AIC
	Weibull	4.88E+6	9.77E+6	9.77E+6
Type Zero	Lognormal	4.88E+6	9.77E+6	9.77E+6
	Exponential	5.20E+6	1.04E+7	1.04E+7
	Weibull	2.74E+6	5.48E+6	5.48E+6
Type One	Lognormal	2.75E+6	5.51E+6	5.51E+6
	Exponential	2.86E+6	5.71E+6	5.71E+6
	Weibull	1.21E+6	2.42E+6	2.42E+6
Type A	Lognormal	1.21E+6	2.43E+6	2.43E+6
	Exponential	1.28E+6	2.55E+6	2.55E+6
	Weibull	1.80E+6	3.59E+6	3.59E+6
Type B	Lognormal	1.81E+6	3.62E+6	3.62E+6
	Exponential	1.84E+6	3.69E+6	3.69E+6

Table S1. Statistics for fitting of the IET of HSBA. The distribution is fitted based on the criteria such as the negative log-likelihood, Akaike information criterion, Bayesian information criterion. Weibull distribution and Lognormal distribution are best fit for each type.

2

4

6 6 6 5
